# The relationship between hamstring strength tests and sprint performance in female Gaelic footballers: A correlation and linear regression analysis

**DOI:** 10.1371/journal.pone.0302901

**Published:** 2024-06-10

**Authors:** Enda Whyte, Siobhán O’Connor, Hannah Tobin Jones, Cian McBride, Aisling O’Flynn, Oisin Quinn, Fearghal Behan

**Affiliations:** 1 School of Health and Human Performance, Dublin City University, Dublin, Ireland; 2 Centre for Injury Prevention and Performance, Ireland; 3 Department of Bioengineering, Imperial College London, London, United Kingdom; 4 Discipline of Physiotherapy, Trinity College Dublin, Dublin, Ireland; Loughborough University, UNITED KINGDOM

## Abstract

**Objectives:**

To investigate the relationships between handheld dynamometer (HHD), isokinetic and Nordic hamstrings exercise (NHE) measurements of knee flexor strength and their association with sprinting performance.

**Design:**

Cross-sectional

**Methods:**

The relationships between HHD (prone isometric, prone break and supine break knee flexor strength tests), isokinetic and NHE peak knee flexor strength measures were examined using Pearson product correlations on 38 female footballers. A linear regression analysis was also performed for each pair of dependent variables (10 and 30 metre sprint times) and independent predictor variables (average relative peak torque for HHD, isokinetic and NHE testing).

**Results:**

There were good correlations between HHD tests (*r* = 0.81–0.90, *p* < 0.001, R^2^ = 0.65–0.82), moderate correlations between HHD and isokinetic peak torque, (*r* = 0.61–0.67, *p* < 0.001, R^2^ = 0.37–0.44) and poor association between the HHD peak torques and isokinetic work (*r* = 0.44–0.46, *p* = 0.005–0.007, R^2^ = 0.20–0.21) and average power (*r* = 0.39–0.45, n = 36, *p* = 0.006–0.019, R^2^ = 0.15–0.22). There was a poor association between NHE peak torque and isokinetic total work (*r* = 0.34, *p* = 0.04, R^2^ = 0.12). No associations between knee flexor strength and sprint times were observed (*p* = 0.12–0.79, *r*^*2*^ = 0.002–0.086).

**Conclusions:**

Moderate to good correlations within HHD testing and poor to moderate correlations between HHD and isokinetic testing were observed. HHD knee flexor torque assessment may be useful to regularly chart the progress of hamstring rehabilitation for female footballers. Knee flexor strength assessments were not associated with sprint times in female footballers. Other aspects of knee flexor strength and sprint performance should be investigated to assist clinicians in making return to running and sprinting decisions in this population.

## Introduction

Hamstring strain injuries (HSI) are a common time loss injury in female field sports with high injury burden [1, 2]. Therefore, rehabilitation and prevention of HSIs in female footballers are of paramount importance to sports medicine clinicians. Almost 70% of HSIs occur during running and sprinting in female football [3] with the hamstrings experiencing the greatest loading during the terminal swing phase of the running cycle [4]. Despite significant research on patterns of hamstring activation during sprinting, the possible injury mechanisms are not clear [5]. One theory is that the injury occurs during terminal swing phase as the hamstrings are eccentrically contracting in a lengthened position [4]. Alternatively, it has been proposed that the hamstrings largely contract isometrically in the swing phase of running [6]. Consequently, a significant amount of research has investigated the relationship between hamstring strength and risk of injury and identified the need for development of progressive, monitored rehabilitation protocols maximise athletes’ potential to safely to return to high performance activities and the addition of hamstring strengthening programmes to regular training to minimise injury risk [7].

Clinicians require the ability to accurately assess hamstring strength to assess for the level of potential injury risk, rehabilitation progression and determination of the appropriate return to training, running, sprinting and ultimately to sport performance [7]. Deficits in hamstring strength measured using hand held dynamometry (HHD) [8, 9], isokinetic dynamometry [10–12] and during the Nordic hamstring exercise (NHE) [13, 14] have been associated with HSI, with the majority of research focussing on male athletes. A number of strength assessments are required during the rehabilitation process typically progressing from isometric assessment in positions of inner hamstring muscle length in the early stage to eccentric strength in the outer ranges of hamstring length in the later stages [7] with specific strength assessments offering prognostic value for return to running [15, 16]. There are several factors that affect the ability of the hamstring muscles to generate force including the different types of contractions (isometric, concentric, eccentric) [17, 18], speed of contraction [17, 19] and hamstring muscle length [18, 20], all which must be considered by the clinician.

There are a number of hamstring muscle assessments that are commonly used in clinical practice that can assess different parameters such as contraction type, muscle lengths and joint velocity. Isokinetic testing is considered the gold standard of muscle strength assessment [21] and can assess isometric strength, and isokinetic strength (concentrically and eccentrically) in different positions of hamstring length providing a range of strength measurement outputs (e.g. torque, work, power and angle of peak torque). However it can be resource and time intensive [22]. HHD offers a cost and time effective option that allows for the assessment of strength in various hamstring muscle lengths [9, 22, 23]and has been found to be reliable and valid compared with isokinetic testing [21]. Furthermore, there is no consensus for HHD testing on the most appropriate testing positions, contraction types [24], or how different HHD methods relate to other strength assessments such as isokinetic dynamometry and during the NHE. Finally, the assessment of eccentric knee flexor strength during the NHE has demonstrated excellent reliability [18], has a range of strength measurement outputs and is time efficient. However, it is limited to assessment of eccentric contraction, it underlies considerable interindividual variations of range of motion and controlled movement speed and can be influenced by exercise execution quality [25]. Therefore, an understanding of the relationships between common methods of hamstring strength assessment could contribute to clinical decision making.

It has been shown that peak eccentric torque measured isokinetically [26, 27] and during the NHE [28, 29] is associated with sprint performance, although this has not been demonstrated with HHD measures of peak torque in males [30]. Other parameters of hamstring muscle strength, such as power and work, are strongly related to sprint performance [31, 32] and associated with risk of hamstring injury in males [12]. Despite the fact that hamstring injuries are common in female footballers [1, 2] during running and sprinting [3], and that females have different hip and knee kinematics and gluteal and hamstring activation levels during high speed running compared with males [33], there has been little investigation into the relationships between different methods of hamstring assessment and sprinting performance in the female population [34].

Therefore, the aims of this study are to investigate the relationship between HHD, isokinetic and NHE strength measurements and to investigate the relationship between hamstring strength measurements and athletic performance (sprint times (10 metres and 30 metres)) in varsity level female Gaelic footballers. We hypothesised that there would be good levels of correlation between hamstring strength when assessed using different methods (HHD, ISOK and during NHE). We also hypothesised that the HHD tests would not be associated with sprint performance, whereas there would be an association between isokinetic and NHE assessments and sprint performance.

## Materials and methods

This exploratory cross-sectional study consisted of two elements: the relationship between HHD measures of hamstring strength, isokinetic and NHE measures; and the association between hamstring strength measures (HHD, isokinetic and NHE) and sprint performance (10m and 30 m sprint times) in female Gaelic footballers. Approval was granted by the local research ethics committee (2020_018_EW_ATT). All participants provided informed consent to take part in the research. Based on an alpha level of 0.05 and power of 0.8 and previous research that investigated the associations between HHD and isokinetic measures (*r* = 0.48 [22]), HHD and NHE measures (*r* = 0.51 [35]) and peak knee flexor torque measures and sprint performance (*r* = 0.50–0.52 [27, 29]) a minimum requirement of 27–32 participants was calculated. To allow for potential dropout, 38 female, varsity Gaelic footballers (age 21.52 ± 2.3 years, height 167.1 ± 5.2cm, weight 68.2 ± 11.3 kg) were recruited. Inclusion criteria were that all participants were varsity level Gaelic footballers, have a history of strength training, participating in training and matches a minimum of 3 times per week and free from any lower limb injuries for the last 3 months. Participants visited the laboratory on three occasions between the 20^th^ of January, 2020 and the 14^th^ of February, 2020. Firstly, they attended a familiarization session followed by two testing sessions, each session separated by 7 days. Participants completed a standardised warm-up during the familiarisation and prior to the testing sessions by cycling on a stationary ergometer between 80 and 100 rpm for 10 minutes [10]. During the first testing session, general health and injury questionnaires were completed by participants and their body height, body mass and chronological age were recorded followed by three HHD knee flexor strength assessments (the isometric prone, prone break and supine break tests). Participants were given a 60-minute break to negate the effects of fatigue before isokinetic dynamometric measures of peak hamstring torque were recorded, preceded by the standardised warm-up. On day 2, NHE hamstring strength testing was completed followed by sprint testing after a 60-minute break. Relative knee flexor peak torque (Nmkg^-1^) for HHD testing, isokinetic eccentric torque (Nmkg^-1^), work (Jkg^-1^) and average power (Wkg^-1^) and peak eccentric torque during NHE testing (Nmkg^-1^) were recorded as relative strength measures have been shown to have greater association with sprint times than absolute measures [29]. Dependent variables were 10m and 30m sprint times (seconds). All hamstring strength data were collected in the university’s athletic therapy facility. Sprint performance data was recorded in a covered out-door tartan track on the university campus. Players wore their regular athletic training shoes for sprint testing. No vigorous physical activity was completed in the 24 hours prior to testing.

### Hand-held dynamometry

Isometric and eccentric knee flexor strength was assessed using a JTECH Commander Echo handheld dynamometer, sampling frequency of 50-60Hz (JTECH Medical, Midvale, UT, USA). Two warm-up repetitions were completed where participants were instructed to complete them at 60% and 80% of maximal effort. Ten second rest intervals were given between each practice repetition and 20 seconds between the final practice and maximal test effort. Participants were directed to use maximum effort, whilst gently holding onto the side of the plinth for stabilisation. Participants were instructed to “Go ahead-push-push-push-push-push and relax”, for each contraction [23]. Each contraction was isometrically resisted for approximately 3 seconds [22] during which the tester observed the HHD display to ensure that the force had plateaued. For the break tests, after the initial 3 seconds and plateauing of the force production, a smooth “break force” was applied by the tester, perpendicular to the shank, that moved the knee through the available range over approximately 1 second. The highest score of three trials was recorded. Leg length was calculated by measuring the distance from the lateral femoral epicondyle to 5cm proximal to the lateral malleolus, where the HHD was positioned. A 15-minute break was given between each of the HHD knee flexor strength assessments (the isometric prone, prone break and supine break tests) to minimise the effects of fatigue. A preliminary intra-tester reliability study demonstrated a high level of reliability for the three HHD tests (ICC 0.89–0.96) (S1 Table).

**Fig 1:**
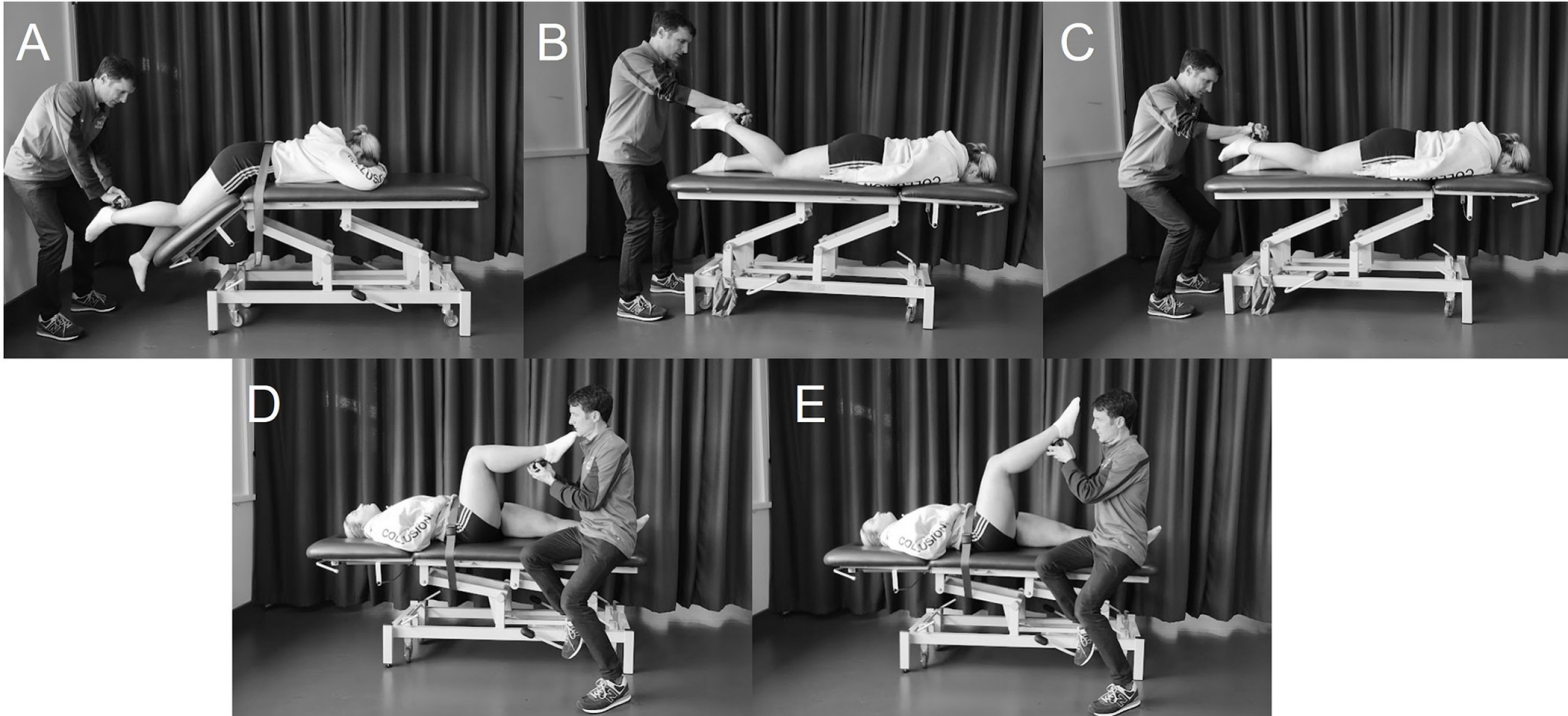
Hand-held dynamometry testing setup. (A) Prone isometric test with hip in 45° flexion and knee in 30° flexion. (B and C) Prone break test. Beginning in 45° knee flexion (B) and pulled through 30° knee range of motion towards an extended position (C). (D and E) Supine break test. Beginning with hip and knee in 90° flexion (D) and pushed through 30° knee range of motion towards an extended position (E).

Three HHD assessment methods were selected to assess knee flexor strength across different combinations of hip and knee positions as this influences the force generating capacity of the hamstrings [36]. The prone isometric test was completed as described by Wollin et al. [23]. It has been found to have good levels of intra tester and inter tester reliability and is proposed to be a position associated with the terminal swing phase of running [23]. Participants lay prone with the hip flexed to 45° and knee flexed to 30°. The prone break test was performed in accordance with Whiteley et al.[22] which has been shown to have excellent intertester reliability [22] and is useful in predicting return to play duration following HSI [16]. Participants lay prone with the hips in neutral and the knee of the limb to be tested flexed to 45°. A ‘break force’ was obtained by pulling down the athlete’s heel to the plinth once the examiner felt the force had peaked. A stabilisation belt was placed at the levels of the ischial tuberosities to minimise unwanted hip movements during testing. Finally, the supine break test was completed as described by Whiteley et al. [37]. It has good to excellent inter and intra tester reliability [37] and is associated with rehabilitation progression following HSI [15, 16]. Participants were positioned in supine and the knee and hip of the tested limb in 90° flexion as confirmed with a goniometer. A stabilisation belt was placed across the ASIS bilaterally and one across the contralateral limb, proximal to the patella. The participant was instructed to keep the hip in the testing position (i.e., in 90° hip flexion), push down against the device for 3s and once the examiner felt the force had peaked, a perpendicular ‘break force’ was applied (see Fig 1). If the participant moved their thigh from the testing position, the test was not considered valid, and the procedure was repeated.

### Isokinetic testing

The Biodex III isokinetic dynamometer (Biodex Medical Systems, Shirley, NY, USA) with a sampling frequency of 100 Hz was used to measure knee flexor eccentric peak torque, work and power. Participants were seated with their hips flexed to 100° and the fulcrum of the dynamometer aligned to the lateral epicondyle of the test leg. The isokinetic range was fixed between 90° to 10° of knee flexion (0° equals full knee extension) at 60°.s^-1^. Calibration was completed as per manufacturer’s instructions, and all torques were corrected for gravity. During the familiarisation session, participants completed submaximal eccentric repetitions at an angular speed of 60°.s^-1^. Participants were instructed to complete the eccentric repetitions at 50% effort and to allow the lower leg to passively return to the starting position with the knee flexed at 90°. Participants completed an average of 5.2 ± 1.3 repetitions before they and the tester were confident in their technique. Prior to testing, two warm-up repetitions were completed at approximately 60% and 80% of perceived maximum effort. Following this, three repetitions of maximal eccentric knee flexor strength at an angular speed of 60°.s^-1^ were completed during which participants were encouraged to promote maximal effort. This was repeated 3 times with 60 second rest in between each series of contractions. Participants were instructed to allow the dynamometer to passively return their lower leg to the starting position (90° knee flexion). The highest peak torque, work and power were recorded. Power was calculated using the product of the moment generated and the preselected angular velocity.

### Nordic hamstring testing

All repetitions were performed on the ‘NordBord’ (Vald Performance, Newstead, Australia) at a sampling frequency of 50 Hz. The device has two separate rigid heel fixations, cushioning for the shank with the shanks positioned 45cm above the ground, which are considered important for high-quality performance of the NHE [25]. Participants knelt on the cushioned padding, allowing partial patellar glide [25]. In addition to the standardised warm-up, three submaximal of the NHE repetitions were completed as warm-up for this bilateral exercise. These consisted of NHEs performed at 50%, 75% and 95% of subjectively rated maximal effort [38, 39]. This was followed by a 1-minute rest before a set of three maximal repetitions of the NHE was completed. Participants were required to assume a kneeling position on the padded area of the testing device and with their ankles secured by the rigid heel fixations, immediately proximal to the malleoli. Participants were requested to maintain their arms across their chest and to lean forwards from the knees, lower their body as slowly as possible through as far a range as possible [38, 39]. If participants moved into hip flexion or lumbar lordosis during the test, corrective feedback was provided by the investigator, that repetition was excluded and repeated. A pole was positioned at the participant’s knee at an angle of 20° flexion to determine if the participant was able to control the movement into the final 10–20° of motion and the need for application of additional resistance [38, 39]. No participants were able to achieve this and therefore additional resistance was not required during testing.

### Assessment of sprint performance

Sprint performance measures were assessed using speed gates (Brower, Draper, UT 84020, USA) which have high reported reliability for 0-10m and 0-30m sprint times (ICC = 0.91–0.99 [40]). They were set up at 0m, 10m and 30m intervals, as described by Krommes et al., [41]. Testing was carried out on a covered, tartan track in the university sports grounds. The participants completed a standardised 10-minute warmup, including a 2-minute jog, lower-limb dynamic stretching, running drills and sub maximal sprint efforts. Athletes assumed a three-point starting position with one hand positioned at a line that represented 0 metres. The first speed gate was positioned at ground level, just in front of the toes of the back leg so that it would be broken at toe-off of the back leg. The remaining speed gates were positioned at a height of 100cm, approximately hip height [30]. Each participant performed the 30-metre sprint test three times with 2-minutes rest between each attempt to minimize the effects of fatigue on performance. The fastest time recorded from the three attempts was used in the analysis.

### Statistical analysis

Data were screened for normal distribution using the Shapiro-Wilk test. The relationship between HHD testing of relative knee flexor peak torque (prone isometric test, prone break test and supine break test), isokinetic peak torque, total work and average power and NHE peak torque was investigated using Pearson product moment correlation coefficient. Preliminary analyses were performed to ensure no violation of the assumptions of normality, linearity and homoscedascticity. Relative HHD peak torque (Nmkg^-1^) for the three HHD knee flexor strength tests were compared to isokinetic relative peak eccentric knee flexor torque (Nmkg^-1^), work done (Jkg^-1^) and power (Wkg^-1^) and relative peak torque NHE (Nmkg^-1^). Averaged limb values were used for testing. Post hoc Holm-Bonferroni correction was applied to correct for family-wise error. Correlations were defined as poor (r < 0.5), moderate (0.5 < r < 0.75) good (0.75 < r < 0.9) and excellent (r > 0.9) [42] and coefficients of determination were subsequently calculated.

A linear regression analysis was used for each pair of dependent variables (10 and 30 metre sprint time) and independent predictor variables (average relative peak torque for prone isometric test, prone eccentric break test, supine break test, isokinetic and NHE testing). The association was calculated as the coefficient of determination (R^2^). Data showed normality of residuals on visual inspection of P-P plots. Effect sizes to determine the strength of association were calculated as *f*^*2*^ *= r*^*2*^*/(1-r*^*2*^*)* and considered trivial (f^2^ < 0.02), low (0.02 ≤f^2^ <0.15), medium (0.15 ≤f^2^ <0.35) and high (f^2^ ≥0.35) [43].

Statistical analysis was performed with IBMM SPSS Statistics for Windows Version 25.0, with the level of significance set at *p* <0.05.

## Results

Strength and performance measures for varsity level, female Gaelic footballers (n = 38) are outlined in S2 Table. There were good correlations between the prone isometric, prone break and supine break tests (*r* = 0.81–0.90, n = 38, *p* < 0.001, R^2^ = 0.65–0.82) moderate positive correlations between the HHD and isokinetic peak torque, (*r* = 0.61–0.67, n = 36, *p* < 0.001, R^2^ = 0.37–0.44) and poor, non-significant, positive correlations between the HHD peak torques and isokinetic work (*r* = 0.44–0.46, n = 36, *p* = 0.005–0.007, R^2^ = 0.20–0.21) and average power (*r* = 0.39–0.45, n = 36, *p* = 0.006–0.019, R^2^ = 0.15–0.22). In relation to NHE measurements, there was only a poor, non-significant, positive correlation between NHE peak torque and isokinetic total work (*r* = 0.34, n = 36, *p* = 0.04, R^2^ = 0.12) (Table 1).

**Table 1:** Pearson product-moment correlation and coefficients of determination between measures of average of knee flexor torque, power and work.

	Prone Hamstring Isometric Test (Nmkg^-1^)	Prone Hamstring Break Test (Nmkg^-1^)	Supine Hamstring Break Test (Nmkg^-1^)	Isokinetic Eccentric Peak Knee Flexor Torque (Nmkg^-1^)	Isokinetic Total Knee Eccentric Flexor Work (Jkg^-1^)	Isokinetic Average Eccentric Power (Wkg^-1^)	NHE Peak Torque (Nmkg^-1^)
Prone Hamstring Isometric Test (Nmkg^-1^)	-	0.87* (0.75–0.93) R^2^ = 0.75	0.90* (0.82–0.95) R^2^ = 0.82	0.61* (0.35–0.78) R^2^ = 0.37	0.45 (0.15–0.68) R^2^ = 0.21	0.39 (0.07–0.64) R^2^ = 0.15	0.36 (0.03–0.61) R^2^ = 0.13
Prone Hamstring Break Test (Nmkg^-1^)		-	0.81* (0.65–0.90) R^2^ = 0.65	0.67* (0.65–0.90) R^2^ = 0.44	0.46 (0.16–0.69) R^2^ = 0.21	0.39 (0.07–0.64) R^2^ = 0.15	0.45 (0.14–0.68) R^2^ = 0.25
Supine Hamstring Break Test (Nmkg^-1^)			-	0.64* (0.39–0.80) R^2^ = 0.41	0.44 (0.13–0.67) R^2^ = 0.20	0.45 (0.14–0.68) R^2^ = 0.22	0.25 (-0.08–0.54) R^2^ = 0.06
Isokinetic Eccentric Peak Knee Flexor Torque (Nmkg^-1^)				-	0.62* (0.38–0.79) R^2^ = 0.38	0.63* (0.39–0.79) R^2^ = 0.40	0.10 (-0.23–0.42) R^2^ = 0.01
Isokinetic Total Knee Eccentric Flexor Work (Jkg^-1^)					-	0.90* (0.81–0.95) R^2^ = 0.81	0.34 (0.01–0.60) R^2^ = 0.12
Isokinetic Average Eccentric Power (Wkg^-1^)						-	0.24 (-0.10–0.53) R^2^ = 0.06
NHE Peak Torque (Nmkg^-1^)							-

*Correlation is significant (2-tailed) post Holm-Bonferroni correction

N Newton, kg kilogramme, BW body weight, Nm Newton metre, J Joule, W Watt.

R^2^ = Coefficient of Determination (percentage)

No significant or meaningful associations between measures of hamstring strength and sprint times were observed (*p* = 0.12–0.79, *r*^*2*^ = 0.002–0.086) (Table 2).

**Table 2:** Associations between dependent performance variables (bold) and independent variables of hamstring muscle strength.

Variables	Regression Coefficient (95% CI)	Explained Variance (r^2^)	Effect size (f^2^)	Strength of association	p-value
**10 Metre Sprint Time**					
Prone Hamstring Isometric Test (Nmkg^-1^)	- 0.063 (-0.184–0.058)	0.040	0.04	Low	0.29
Prone Hamstring Break Test (Nmkg^-1^)	- 0.051 (-0.211–0.109)	0.015	0.015	Trivial	0.53
Supine Hamstring Break Test (Nmkg^-1^)	-0.037 (-0.144–0.071)	0.017	0.017	Trivial	0.49
Isokinetic Eccentric Peak Knee Flexor Torque (Nmkg^-1^)	0.028 (-0.062–0.119)	0.013	0.013	Trivial	0.53
Isokinetic Total Knee Eccentric Flexor Work (Jkg^-1^)	0.050 (-0.052–0.152)	0.032	0.033	Low	0.33
Isokinetic Average Eccentric Power (Wkg^-1^)	0.051 (-0.094–0.196)	0.017	0.017	Trivial	0.48
NHE Peak Torque (Nmkg^-1^)	-0.087 (-0.200–0.025)	0.083	0.091	Low	0.12
**30 Metre Sprint Time**					
Prone Hamstring Isometric Test (Nmkg^-1^)	- 0.128 (-0.332–0.076)	0.056	0.059	Low	0.21
Prone Hamstring Break Test (Nmkg^-1^)	- 0.136 (-0.405–0.133)	0.037	0.038	Low	0.31
Supine Hamstring Break Test (Nmkg^-1^)	-0.087 (-0.268–0.095)	0.033	0.034	Low	0.37
Isokinetic Eccentric Peak Knee Flexor Torque (Nmkg^-1^)	0.020 (-0.134–0.175)	0.002	0.002	Trivial	0.79
Isokinetic Total Knee Eccentric Flexor Work (Jkg^-1^)	0.055 (-0.120–0.230)	0.013	0.013	Trivial	0.53
Isokinetic Average Eccentric Power (Wkg^-1^)	0.051 (-0.196–0.298)	0.006	0.006	Trivial	0.68
NHE Peak Torque (Nmkg^-1^)	-0.151 (-0.342–0.040)	0.086	0.095	Low	0.12

N Newton, kg kilogramme, BW body weight, Nm Newton metre, J Joule, W Watt.

## Discussion

The aim of this study was to investigate the relationship between HHD, isokinetic and NHE knee flexor strength measurements and their associations with sprint performance. Our hypothesis that there would be good levels of correlation between hamstring strength measures was not supported as in general, we found moderate to poor correlations between the HHD and isokinetic measurements, and poor to no correlations between isokinetic and NHE measurements. In relation to our hypothesis on the association between measures of knee flexor strength (HHD, isokinetic and NHE) and sprint performance as measured by time, the results of this study supported the hypothesis that the HHD tests would not be associated with sprint performance, whereas the results did not support our hypothesis that there would be an association between isokinetic and NHE assessments and sprint performance.

The good to excellent levels of correlation between the three strength tests using the HHD (*r* = 0.81–0.90) may provide reassurance for clinicians who are required to adapt their strength assessments during the rehabilitation process, moving from isometric, inner to mid-range assessments in the early stages to eccentric, outer range assessment in the later stages of rehabilitation [7]. This may be particularly relevant for clinicians as the supine break test is associated with rehab progression and perceived ease of running [15], mid-range HHD testing can be prognostic for return to running [16] and can predict HSI reinjury [9] and lower peak eccentric force during the prone break test can predict HSI [8]. Therefore, the results of the current study suggest that the HHD testing employed could be used in a practical way to assess the progress of rehabilitation, given sufficient intra-rater ad inter-rater reliability.

In relation to the association between HHD and isokinetic assessments, our study found significant moderate correlations between isokinetic peak knee flexor torque and HHD measures of knee flexor peak torque (prone break test *r* = 0.61; prone hamstring break test *r* = 0.67; and supine break test *r* = 0.64) in female footballers. These are similar to findings for male footballers reported for the prone break test (*r* = 0.54-.61) [22] and stronger than that reported for the isometric outer range hamstring strength test in sitting (r = 0.48–0.53) [24]. Higher correlations have been found when comparing isometric strength testing using a HHD and an isokinetic device (*r* = 0.79–0.87) [24, 44]. However, peak eccentric hamstring action may be more ecologically valid as it potentially plays a major role for the terminal swing phase during sprinting [32]. There were non-significant poor correlations between isokinetic work (*r* = 0.44–0.46) and power (*r* = 0.39–0.45) measurements and the HHD assessments which explains between 20–21% and 15–22% of the shared variance respectively. The findings of the current study may be useful for the clinician in the return to play decision making process. Although correlation does not imply a cause and effect relationship, clinicians may decide that moderate correlations between the peak torque isokinetic measurements and HHD measurements may inform their practice: as the HHD assessment has been reported to be more time efficient than isokinetic testing [22], HHD assessments may be an option for a clinician to use for reassessments whereas HHD measurements are not sufficient for clinicians to determine risk of injury compared to isokinetic peak eccentric torque assessments. Additionally, the poor relationships between HHD and isokinetic power and work assessments may not be sufficiently strong for clinicians to use HHD assessment in place of using these isokinetic assessments of work and power to determine risk of injury [12] or sprinting capacity [32, 45].

The current study demonstrated a non-significant poor correlation between NHE testing and the prone isometric (*r* = 0.36), prone break (*r* = 0.45) and supine break test (*p* = 0.14, r = 0.25) for normalised peak torque in female Gaelic footballers. These are similar to Moreno-Perez et al., [35] that investigated the relationship between NHE strength testing and HHD isometric testing in males. They found a significant moderate correlation (*r* = 0.51*)* for the mid-range test (prone isometric hamstring in 15° knee flexion), but only for the non-dominant limb. Similar to the current study, they did not observe a correlation in the outer range test (standing with hip in 90° and knee in 20° flexion) [35]. The outer range tests used by Moreno-Perez et al., [35] and the supine break test used in the current study place the hamstring in a lengthened position with hip flexion, whereas the hamstring is in a shorter position with the hip held in neutral throughout the NHE. Furthermore, Wiesinger et al., [20] found that different modes of eccentric strength assessment produce different results, even when controlling for hip position. Consequently, the different tests assess different hamstring muscle function. Similar to previous research in male athletes [20, 46, 47], the current study found poor corelations between peak torque isokinetic torque and NHE peak torque (*r* = 0.10). Additionally, we found no significant or meaningful correlations between peak eccentric knee flexor torque during the NHE and peak eccentric knee flexor isokinetic power (*r* = 0.12) and peak isokinetic knee flexor work (*r* = 0.34). This may be at least partially explained by the different hamstring activation patterns observed between these assessments [48]. The poor and absent corelations between NHE peak torque measurements and both HHD and isokinetic testing suggest that HHD or isokinetic testing should not be used as an alternative to NHE testing in female Gaelic footballers.

Given the high re-injury rates for HSIs [49, 50], the return to sport, and in particular return to sprinting, is a challenging decision for the clinician. There is a strong relationship between knee flexor eccentric peak force, work and power in the late swing phase and sprint performance when assessed using 3-d musculoskeletal modelling [32] and isokinetically [31]. Given that deficits in knee flexor strength for a variety of assessment methods have been associated with risk of HSI [8–14], it is not surprising that strength assessment is considered an essential element for this decision making [7]. However, a recent Delphi consensus statement found a lack of consensus on specific hamstring strength tests to guide return to running and subsequent sprinting and return to sport [7]. The current study did not find a relationship between any of the hamstring strength measures (HHD, isokinetic or NHE) and sprint performance (10metre and 30 metre sprint times). Individual tests only explained between 1 and 9% of the variance observed in sprint times. These findings indicate that individual hamstring strength assessments are not useful in determining sprint performance as measured by time in this cohort of female Gaelic footballers. Similar to Ishoi et al.[30], we did not find a relationship between isometric knee flexor torque using a HHD and sprint times. We also did not find a relationship with the supine and prone break tests and sprint times indicating that HHD measurements are not useful clinically to assist to determine sprint performance based on sprint times in female Gaelic footballers. The results from the current study conflict with previous research that demonstrated positive correlations between sprint performance and peak eccentric isokinetic force, work and power [26, 27, 31]. This may be partially explained by the fact that the studies by Anderson et al. [26] and Alt et al. [31] conducted mid-range isokinetic assessment of knee flexor function in prone with the hip in neutral in contrast to our testing method of seated, outer range knee flexor assessment. Finally, our study also did not find a relationship between NHE peak knee flexor strength and sprint performance, similar to that reported by Suaraez- Arrones et al. [51], but in contrast to results from Markovic et al. [29] and Jiang et al. [28]. Markovic et al. [29] examined the relationship in adolescent male footballers over 20 metres, which may explain the contradictory findings. When separated by sex, Jiang et al. [28] did not find a significant association between sprint performance and NHE knee flexor peak torque in female volleyball players. The studies by Jiang et al. [28] and Markovic et al. [29] suggest that there might be sex differences in the association between measures of hamstring strength and sprinting, highlighting the importance of research specifically on females with respect to methods of hamstring measurement [34].

The absence of an association between measures of knee flexor strength and sprint performance in females in the current study may be explained by several reasons. Sprint performance is dependent on a range of factors, not just knee flexor strength [45]. It may be that the hamstrings contribute to sprint performance differently in females compared to males, as has been observed in walking and jogging [52]. The positioning of the speed gates will affect the calculation of sprint times and therefore influence the findings of this aspect of the study. In relation to the placement of the initial speed gate in front of the participants’ back foot, different technique may result in changes in overall sprint time. Subsequent speed gates were placed at 100cm height which was considered to be approximately hip height [30]. However, this was not adjusted for each participant and may lead to slightly different spilt times based upon differences in participant height. Also, the methods of strength assessment (peak torque, peak work and peak power) and/or the assessment of sprint performances (10 and 30 metre sprint times) were not sensitive enough in this study on female athletes. Although Ishoi et al. [30] did not find a relationship with peak torque and sprint performance, they did find positive associations between rate of torque development and mechanical sprint variables (maximal horizontal force productions and maximal horizontal power output) which likely explain the negative association they observed between rate of torque development and sprint times. Similarly Prince et al. [53] found moderate correlations between NHE strength and horizontal force production in sprinting. Therefore, future research should more comprehensively investigate the relationship between different aspects of strength testing (e.g. rate of torque development) across the different modes (HHD, isokinetic and NHE) and sprint performance, including mechanical sprint variables in females specifically. This may provide greater evidence to support the clinicians in return to running, sprinting and sport decision making for female footballers.

There are a number of limitations to the current study. Firstly, we looked at only three methods of hamstring HHD testing in the mid and outer ranges. Other assessments, such as the prone isometric test at 15° knee flexion with hip in neutral [9], which has been associated with future injury risk, inner range prone, isometric hamstring strength testing, which has been found to relate to prognosis following hamstring injury [16] or seated isometric hamstring strength testing [24]. Secondly, we just looked at peak torque measurements for HHD and NHE measures and we did not correct for lower leg moment of inertia or correction due to gravitational forces in the HHE assessments. Other measurements such as rate of peak force development, force at set time or impulse based metrics may provide additional information that may be associated with sprinting performance and inform readiness for sprinting decision making. In terms of quality of the NHE, we only partially met the Assessing Nordic Hamstrings Exercise Quality scale [25]. Greater standardisation of the speed of the NHE and recording of the range of the movement, would improve the overall quality of the NHE assessment. We calculated isokinetic power based upon the preselected angular velocity of the dynamometer rather than the actual knee joint angular velocity which has been found to lead to overestimation of power measurements [54]. Finally, we only assessed sprint times to determine sprint performance which is an outcome measure of sprint performance rather than a contributing factor. The recording and analysis of a higher number of split times may also provide a greater level of understanding. Recent studies have demonstrated the relationship between hamstring strength measures and different biomechanical variables that contribute to sprint performance [28, 30].

## Conclusion

The prone isometric, prone break and supine break tests using HHD are highly reliable methods of assessing hamstring strength, correlate highly with each other and have the potential to be both clinically practical and prognostic in the management of female footballers. HHD testing may be used judiciously during clinical reassessments in lieu of isokinetic peak torque assessments, but they should not be used in place of isokinetic knee flexor and work or NHE strength assessments or criterion based isokinetic testing. The peak torque, work and power assessments conducted in the current study do not correlate with sprint times in the female footballers of the current study and should not be used as a proxy method to determine ability to sprint. More detailed strength measures, such as rate of force development, and sprint metrics, such as maximal horizontal force and power, may provide greater direction for clinicians dealing with female footballers.

## Supporting information

S1 Data (XLSX)

S1 File (XLSX)

S1 Table Intra rater reliability of the prone isometric, prone break and supine break tests. (DOCX)

S2 Table Strength and performance measurements for university, ladies gaelic footballers. (DOCX)
