# Supplementary material for: The relationship between hamstring strength tests and sprint performance in female Gaelic footballers: A correlation and linear regression analysis
Source: PLoS One. 2024 Jun 10;19(6):e0302901. doi: 10.1371/journal.pone.0302901 (PMC11164353; doi:10.1371/journal.pone.0302901)
Supplement: S1 Table — (DOCX) [file pone.0302901.s003.docx]

**Supplementary Table 1 Intra rater Reliability of the Prone Isometric, Prone Break and Supine Break Tests**

| **Test** | **Leg** | **Time 1** | | **Time 2** | **Paired t Test** | | **ICC (CI 95%)** | | **SEM** | | **SEM%** | | **MDC** | | **MDC%** | |  |
| --- | --- | --- | --- | --- | --- | --- | --- | --- | --- | --- | --- | --- | --- | --- | --- | --- | --- |
|  |  | **Mean (SD)** | **Mean (SD)** | | | ***p* Value** | |  | |  | |  | |  | |  | |
| **Prone isometric (N)** | ND | 235.64 (98.21) | 255.46 (94.53) | | | Ns | | 0.94 (0.82 -0.98) | | 23.33 | | 9.5 | | 64.67 | | 26.3 | |
|  | D | 245.55 (91.28) | 249.91 (83.33) | | | Ns | | 0.95 (0.86 - 0.98) | | 19.21 | | 7.8 | | 53.25 | | 21.5 | |
|  | Average | 240.60 (88.97) | 252.63 (86.74) | | | Ns | | 0.96 (0.89 - 0.99) | | 17.31 | | 7.0 | | 47.98 | | 19.5 | |
| **Prone break (N)** | ND | 220.26 (93.86) | 224.24 (91.67) | | | Ns | | 0.95 (0.85 - 0.98) | | 20.38 | | 9.2 | | 56.50 | | 25.5 | |
|  | D | 228.81 (77.99) | 215.89 (70.65) | | | Ns | | 0.89 (0.69 - 0.96) | | 24.35 | | 11.0 | | 67.49 | | 30.4 | |
|  | Average | 224.53 (84.90) | 219.06 (77.91) | | | Ns | | 0.94 (0.84 - 0.98) | | 19.62 | | 8.8 | | 54.39 | | 24.5 | |
| **Supine break (N)** | ND | 273.60 (98.26) | 271.04 (100.69) | | | Ns | | 0.95 (0.84 - 0.98) | | 21.86 | | 8.0 | | 60.59 | | 22.2 | |
|  | D | 263.53 (81.63) | 275.47 (97.08) | | | Ns | | 0.93 (0.79 - 0.97) | | 23.37 | | 8.7 | | 64.78 | | 24.0 | |
|  | Average | 268.57 (88.86) | 273.26 (96.31) | | | Ns | | 0.95 (0.86 - 0.98) | | 20.37 | | 7.5 | | 56.45 | | 20.8 | |

N newton, ICC intraclass correlation coefficient, CI confidence interval, SD standard deviation, SEM standard error of measurement, MDC minimal detectable change, % percentage, ns non-significant, ND non-dominant, D dominant.
