# Supplementary material for: The relationship between hamstring strength tests and sprint performance in female Gaelic footballers: A correlation and linear regression analysis
Source: PLoS One. 2024 Jun 10;19(6):e0302901. doi: 10.1371/journal.pone.0302901 (PMC11164353; doi:10.1371/journal.pone.0302901)
Supplement: S2 Table — (DOCX) [file pone.0302901.s004.docx]

**Supplementary Table 2. Strength and Performance Measurements for University, Ladies Gaelic Footballers (n=38)**

| **Measurements** |  | **Non-Dominant Limb** | | **Dominant Limb** | | **Average** | |
| --- | --- | --- | --- | --- | --- | --- | --- |
| Handheld Dynamometry | Prone Hamstring Isometric Test Peak Torque (Nmkg^-1^) | 1.46 | 0.35 | 1.42 | 0.40 | 1.44 | 0.35 |
|  | Prone Hamstring Break Test Peak Torque (Nmkg^-1^) | 1.16 | 0.32 | 1.16 | 0.28 | 1.16 | 0.27 |
|  | Supine Hamstring Break Test Peak Torque (Nmkg^-1^) | 1.47 | 0.42 | 1.56 | 0.48 | 1.51 | 0.41 |
| Isokinetic | Eccentric Peak Torque (Nmkg^-1^) | 2.35 | 0.43 | 2.60 | 0.54 | 2.47 | 0.44 |
|  | Total Eccentric Work (Jkg^-1^) | 2.47 | 0.39 | 2.67 | 0.46 | 2.57 | 0.40 |
|  | Average Eccentric Power (Wkg^-1^) | 1.70 | 0.29 | 1.80 | 0.30 | 1.75 | 0.28 |
| Nordic Hamstring Exercise | Peak Torque (Nmkg^-1^) | 1.53 | 0.30 | 1.52 | 0.47 | 1.52 | 0.36 |
| Performance | 10 metre sprint time (s) |  |  |  |  | 2.05 | 0.11 |
|  | 30 metre sprint time (s) |  |  |  |  | 4.98 | 0.19 |

N Newton, kg kilogramme, BW body weight, Nm Newton metre, J Joule, W Watt, s second.
